# Feasibility and potential diagnostic value of [^18^F]PI-2620 PET in patients with down syndrome and Alzheimer’s disease: a case series

**DOI:** 10.3389/fnins.2024.1505999

**Published:** 2025-01-06

**Authors:** Olivia Wagemann, Matthias Brendel, Nicolai Franzmeier, Georg Nübling, Johannes Gnörich, Mirlind Zaganjori, Catharina Prix, Anna Stockbauer, Elisabeth Wlasich, Sandra V. Loosli, Katja Sandkühler, Lukas Frontzkowski, Günter Höglinger, Johannes Levin

**Affiliations:** ^1^Department of Neurology, University Hospital, Ludwig-Maximilians-University (LMU) Munich, Munich, Germany; ^2^German Center for Neurodegenerative Disease (DZNE), Munich, Germany; ^3^Department of Nuclear Medicine, University Hospital, Ludwig-Maximilians-University (LMU) Munich, Munich, Germany; ^4^Institute for Stroke and Dementia Research (ISD), University Hospital, LMU Munich, Munich, Germany; ^5^Department of Psychiatry and Neurochemistry, Institute of Neuroscience and Physiology, The Sahlgrenska Academy, University of Gothenburg, Mölndal and Gothenburg, Sweden; ^6^Munich Cluster for Systems Neurology (SyNergy), Munich, Germany; ^7^Department of Neurology, University Hospital Zurich, Zurich, Switzerland

**Keywords:** down syndrome, Alzheimer, tau PET, 18F-PI-2620, case series, trisomy 21

## Abstract

**Purpose of the report:**

Adults with Down Syndrome (DS) have a substantially increased risk for Alzheimer’s disease (AD) due to the triplicated amyloid-precursor-protein gene on chromosome 21, resulting in amyloid and tau accumulation. However, tau PET assessments are not sufficiently implemented in DS-AD research or clinical work-up, and second-generation tau tracers such as [^18^F]PI-2620 have not been thoroughly characterized in adults with DS. We aim at illustrating feasibility and potential diagnostic value of tau PET imaging with [^18^F]PI-2620 for the diagnosis of DS-AD.

**Materials and methods:**

Five adults with DS (40% female, aged 43–62) and cognitive decline underwent clinical assessments, neuropsychological testing, lumbar puncture and multimodal neuroimaging. All underwent [^18^F]PI-2620 tau PET. Visual read of tau PET scans was performed by three blinded raters, assessing increased tracer uptake in brain areas corresponding to the six Braak stage regions and basal ganglia.

**Results:**

Visual read of tau burden revealed three tau-positive individuals which corresponded to their clinical decline while two cognitively stable individuals were rated as negative. Rating showed high inter-rater reliability for all Braak stages.

**Conclusion:**

Tau PET imaging is a feasible and important biomarker assessment in the differential diagnosis of cognitive decline in adults with DS at risk of developing AD.

## Introduction

Down syndrome (DS) is the most common chromosomal aberration worldwide (Fortea et al., 2021) caused by the triplication of chromosome 21 which harbors the *amyloid precursor protein* gene (*APP*; Doran et al., 2017), resulting in an increased gene dosage causing *APP* overexpression and consequently the accumulation of beta-amyloid in the central nervous system (Oyama et al., 2008). Following the deposition of amyloid plaques, hyperphosphorylation of intracellular tau protein is initiated, leading to changes in its metabolism and ultimately to the formation of neurofibrillary tau tangles (NFT; Selkoe and Hardy, 2016). These processes are considered major hallmarks of Alzheimer’s disease (AD), further leading to synaptic dysfunction, neuroinflammation, neuronal death and cognitive as well as functional decline (Selkoe and Hardy, 2016).

Therefore, adults with DS have a ~ 90% risk of developing clinical symptoms of AD by the age of 65(McCarron et al., 2017), rendering AD their main cause of death (Hithersay et al., 2019). Yet, due to the inter- and intraindividual variability in intellectual disability (ID) as well as the heterogeneity in clinical symptoms (Beresford-Webb et al., 2021; Videla et al., 2022), the clinical diagnosis of AD in DS (DS-AD) remains challenging and is often misdiagnosed as a psychiatric disorder, delaying appropriate treatment with acetylcholinesterase inhibitors or an n-methyl-d-aspartate inhibitor as well as a timely initiation of supportive and social measures with high relevance to their everyday lives.

In this context, the paradigm shift towards a biological definition of AD, supported by the A/T/N-framework (Dubois et al., 2021; Jack et al., 2018) is of high value for this population. These criteria aim to assess the absence (“-“) or presence (“+”) of pathophysiological correlates of cerebral amyloid (“A”) accumulation, intracellular hyperphosphorylation of tau protein (“T”) and general signs of neuronal injury or neurodegeneration (“N”; Jack et al., 2016). Considering that in adults with DS, first amyloid plaques form early into the third decade, with NFT following around the age of 35(Fortea et al., 2021), there is considerable promise in applying the A/T/N framework to facilitate the diagnostic process and increase diagnostic certainty for cognitive decline due to AD. This is pivotal since the US Food and Drug Administration (FDA) granted full approval to the anti-amyloid drug lecanemab (van Dyck et al., 2023) in July 2023 (FDA news release July 06, 2023) and donanemab in July 2024 (FDA news release July 02, 2024), underlining the imperative of including this overlooked and vulnerable population in future interventional trials (Strydom et al., 2018).

In recent years, positron emission tomography (PET) has emerged as promising tool for visualizing and quantifying topological disseminations of tau pathology *in vivo* (Ossenkoppele and Hansson, 2021). First generation radiotracers such as [^18^F]AV-1451 and [^18^F]THK-5351 showed high correspondence with tau pathology distribution at autopsy as well as cognitive performance (Smith et al., 2019; Bucci et al., 2021; Sperling et al., 2018) and performed well at differentiating AD from other non-AD-tauopathies (Ossenkoppele et al., 2018). However, insufficient tracer specificity, resulting in significant off-target binding at pigment-containing and vascular structures, meninges and monoaminoxidases A and B, remained a major concern (Lemoine et al., 2020). Since, second-generation ligands such as [^18^F]PI-2620 have been developed, providing increased affinity for tau and considerably less off-target binding (Kroth et al., 2019). In line with tau distribution patterns in sporadic AD (Braak and Braak, 1991), autopsy investigations of DS have found tau pathology to affect the entorhinal cortex first, then the hippocampus as well as the locus coeruleus and dorsal raphe nucleus, eventually reaching the neocortical areas in the fifth decade (Davidson et al., 2018; Mann et al., 1986).

With this case-series, we aim to illustrate the feasibility and potential diagnostic value of tau PET imaging using a second-generation tracer for the diagnosis of DS-AD in a clinical setting.

## Methods

### Patients and clinical work up

All cases were referred to our department of neurology at the university hospital due to a suspected cognitive decline and are part of a monocentric study investigating AD in adults with DS, for which each individual or their respective legal proxy provided informed written consent. The study is approved by the local ethics committee (vote #17-126) and conducted in accordance with the Declaration of Helsinki.

For each patient, chromosome analyses assessed the type of trisomy 21. ID was stratified according to the criteria of the Diagnostic and Statistical Manual of Mental Disorders, 5th edition (DSM-V; American Psychological Association, 2022) into mild, moderate, severe, or profound based on the individuals’ best-ever level of functioning according to detailed interviews with caregivers, neuropsychological assessment, behavioral observation, and review of medical records.

For all patients, cognitive assessments were carried out by trained neuropsychologists using the validated German version of the Cambridge Cognitive Examination for Older Adults with Down Syndrome (CAMCOG-DS) assessing the individual performance on orientation, language, memory, praxis, abstract thinking, attention and visual perception (Beresford-Webb et al., 2021; Nübling et al., 2020; Loosli et al., 2024). Diagnostic blood and, for some, cerebral spinal fluid (CSF) samples were collected and analyzed.

Tau PET data was acquired between February 2019 and October 2021 at the department of nuclear medicine at our University Hospital. All PET scans were performed with the clinical suspicion of progressive cognitive and/or functional decline using the investigational [^18^F]PI-2620 tracer (Life Molecular Imaging Technologies, Inc., Germany). After intravenous injection of 185 ± 10 MBq [^18^F]PI-2620 tracer, PET imaging was performed in a full dynamic 0–60 min setting, preceded by a low-dose CT scan for attenuation correction. For PET, dynamic emission recordings were framed into 6 × 30 s, 4 × 60 s, 4 × 120 s, and 9 × 300 s. PET data were reconstructed iteratively with a matrix size of 336 × 336 × 109/ 400 × 400 × 148, a voxel size of 1.018 × 1.018 × 2.027/1.018 × 1.018 × 1.500 mm^3^/and a slice thickness of 2.027/1.500 mm and standard corrections were applied. Further, tau PET assessments from sex-matched healthy controls (HC) without cognitive impairment were included as reference, each of these participants provided written consent.

### Visual rating of tau PET images

Three physicians specialized in the field of nuclear medicine (MB, JG, MZ) and blinded to the patients’ symptoms and clinical diagnoses were asked to rate all five DS tau PET scans, and five scans from euploid HC, independently. A scan was considered tau positive when tracer uptake was rated as mildly (1), moderately (2) or markedly (3) elevated. For rating, the distribution pattern of cortical tracer binding was assessed in six areas according to Braak staging (Braak and Braak, 1991), and the basal ganglia as seventh region of interest. By adjusting the color scale, the predominant color in the inferior cerebellar cortex was set to 1 to function as reference. A final consensus was defined as a 2/3 majority for each Braak-stage region and the basal ganglia for every patient.

### Statistical analysis

Statistical analyses were performed using R (version 4.1.3; R Core Team, 2021). For inter-rater reliability between the three raters, Fleiss’s kappa was assessed as average of all possible two-rater’s kappa (Conger, 1980).

## Results

### Case 1

A 51-year-old male (Table 1) presented with increasing memory problems over the past 6 months, forgetting birthdays, names and telephone numbers while also engaging less in social interactions. A recent MRI reported a mesiotemporal brain atrophy with subtle enlargement of the subarachnoid space. The neurological examination showed no abnormalities, the neuropsychological assessment revealed a performance score on the CAMCOG-DS in the medium range (68/108) compared to other adults with DS, with individual weaknesses in memory, attention and a task testing apraxia. CSF analysis showed a decreased amyloid ratio (Aβ42/40) ratio, without changes in phosphorylated tau 181 (pTau-181) and total tau (tTau). The A/T/N-score therefore was summarized as A + T-N+ and, due to a reported cognitive decline that so far had not impacted daily functions, the diagnosis of a mild cognitive impairment with early-stage correlates of AD pathology was reached and symptomatic treatment with donepezil 5 mg was initiated.

**Table 1:** Characteristics of DS patients included in this case series.

Case	1	2	3	4	5
Sex	M	M	F	F	M
Type of trisomy 21	Full	Full	Full	Full	Full
Intellectual disability (best level)	Mild	Mild	Mild	Moderate	Moderate
Chronic diseases	Hypothyreoidism, sleep apnea	Hypothyreoidism, epilepsy, tinnitus, right bundle branch block	Hypothyreoidism, bradycardia, myoclonia of unknown etiology	Hypothyreoidism, asthma bronchiale, hepatopathy, status post ards due to covid19 pneumonia, status post acute kidney failure	Hypothyreoidism, bilateral cataract, prebyacusis
Age at symptom onset	51	53	39	52	60
Age at baseline	51	60	42	53	62
CAMCOG-DS at baseline	68/108	65/109	23/109	DNA	DNA
Age at tau PET	56	61	43	53	62
CAMCOG-DS at time of tau PET	69/108	51/109	NA	64/109	62/109
Qualitative majority read tau PET*					
Braak I— III	0	3	3	3	0
Braak IV	0	2	3	3	0
Braak V	0	2	3	3	0
Braak VI	0	2	3	3	0
Basal Ganglia	1	3	1/0/2	2/0/3	1

*Tau positivity was qualitatively assessed by three blinded raters as tracer uptake in the respective region being not (0), mildly (1), moderately (2) or markedly (3) elevated. If a majority consensus could not be reached, the rating of each rater is listed. NA = not available. DNA = does not apply since baseline assessment was at time of tau PET.

Over the course of 3 years, it was reported that memory impairment had worsened and that support for daily life activities (e.g., showering) had become necessary. However, the cognitive performance at the recurring clinical visits remained stable (52 years: 68/109, 54 years: 69/109) and a Florbetaben-PET at 54 found no signs of cortical amyloid plaques. At the age of 56 years, an additional tau-PET was performed without any compliance issues, with the official report finding no cortical increase of [^18^F]PI-2620 and only mild tracer enrichment in the bilateral striatum.

### Case 2

A 60-year-old male presented with a 7-year history of progressive memory problems, resulting in frequent misplacement of belongings and struggling to remember recent events. He had become dependent on other people’s support in everyday life and developed behavioral abnormalities like collecting random objects. In addition, his day-night rhythm was disturbed, causing him to be very active at night. In the neurological assessment, no abnormalities were seen. His performance on the CAMCOG-DS amounted to a score of 65/109. For further differential diagnosis, a Florbetaben-PET was performed, showing elevated tracer uptake in the posterior cingulate cortex as well as the frontal, parietal and temporal cortex. The diagnosis of clinical dementia with probable AD (A + T?N?) was reached and donepezil 5 mg was prescribed.

On 1 year follow-up, the cognitive performance had worsened (51/109). Therefore, a tau-PET was performed, reporting moderate [^18^F]PI-2620 enhancement in the precuneus and posterior cingulate cortex of both sides, as well as a mild enhancement in the parietal and lateral temporal cortices of both sides, confirming AD (A + T + N?). Further, strong [^18^F]PI-2620 enhancement was observed in the bilateral striatum.

### Case 3

A 42-year-old female presented with progressive cognitive decline over the past 3 years with increasing difficulty in word-finding, reading, and writing as well as being more withdrawn and emotional, resulting in frequent crying and anxiety. She also showed general motoric slowing due to which she had been reassigned within her workplace. The neurological assessment remained unremarkable; however, the cognitive examination using the CAMCOG-DS revealed deficits in all areas examined (23/109). Blood analysis showed no abnormalities, and the clinical diagnosis of dementia was reached.

During a follow up visit 12 months later, further differential diagnostics was carried out, with CSF analysis showing slightly decreased Aβ42 levels but normal levels for Aβ42/40 ratio, pTau-181 and tTau. Meanwhile, a cCT reported general brain atrophy.

To assess fibrillar tau pathology, a tau PET was performed with good compliance from the patient. Here, imaging revealed strong [^18^F]PI-2620 enhancement in the mesial temporal, lateral temporal, parietal and frontal cortices of both sides. Also, moderate striatal [^18^F]PI-2620 enrichment was observed. Reaching the diagnosis of AD (A + T + N+), donepezil 10 mg and memantine 5 mg were prescribed.

### Case 4

A 53-year-old female visited our clinic due to increasing forgetfulness first noticed approximately 9 months ago. Temporal and spatial orientation was reportedly impaired, and she often forgot instructions, resulting in her leaving her work place a couple months prior. In addition, she had become more anxious and often worried about being left alone. The neurological status was without abnormalities. Her cognitive performance on the CAMCOG-DS was set in the medium range (64/109), with distinct deficits in memory function. Due to cognitive deficits, reportedly impacting the patient in everyday life, she was clinically diagnosed with dementia.

To investigate the underlying cause, lumbar puncture and tau PET were performed without complications, with the former revealing a decreased Aβ42/40 ratio as well as elevations of pTau-181 and tTau levels in the CSF. The tau imaging report found strong cortical [^18^F]PI-2620 enhancement in the mesial temporal, lateral temporal, parietal and frontal cortices of both sides, each with emphasis on the left side. Moreover, moderate [^18^F]PI-2620 enhancement was seen in the caudate nucleus. With the diagnosis AD (A + T + N+), medication with donepezil 5 mg was initiated.

### Case 5

The 62-year-old male presented with progressive forgetfulness and a word-finding disorder over the course of 2 years. In addition, he seemed more emotional, with frequent outbursts of anger, especially when feeling overwhelmed in everyday life. He slept a lot during the day and had had to switch to part-time employment.

While the neurological examination remained inconspicuous, the cognitive assessment revealed distinct deficits in praxia, memory and orientation with a total score of 62/109 in the CAMCOG-DS. In context of the reported progressive functional decline, a clinical dementia syndrome was diagnosed.

For further etiological differential diagnosis, lumbar puncture and tau PET were sought, but due to compliance issues, only the latter was performed. Here, tau imaging reported mild [^18^F]PI-2620 enhancement in the temporal and parietal cortex while the basal ganglia showed moderate symmetric [^18^F]PI-2620 enrichment. Medication with rivastigmine, which had already been described by the general physician, was continued. Upon a follow-up visit 1 year later, the patient was reported to have shown further functional decline but showed comparable performance on the CAMOG-DS.

### Visual [18F]PI-2620 tau-PET rating

**Figure 1:**
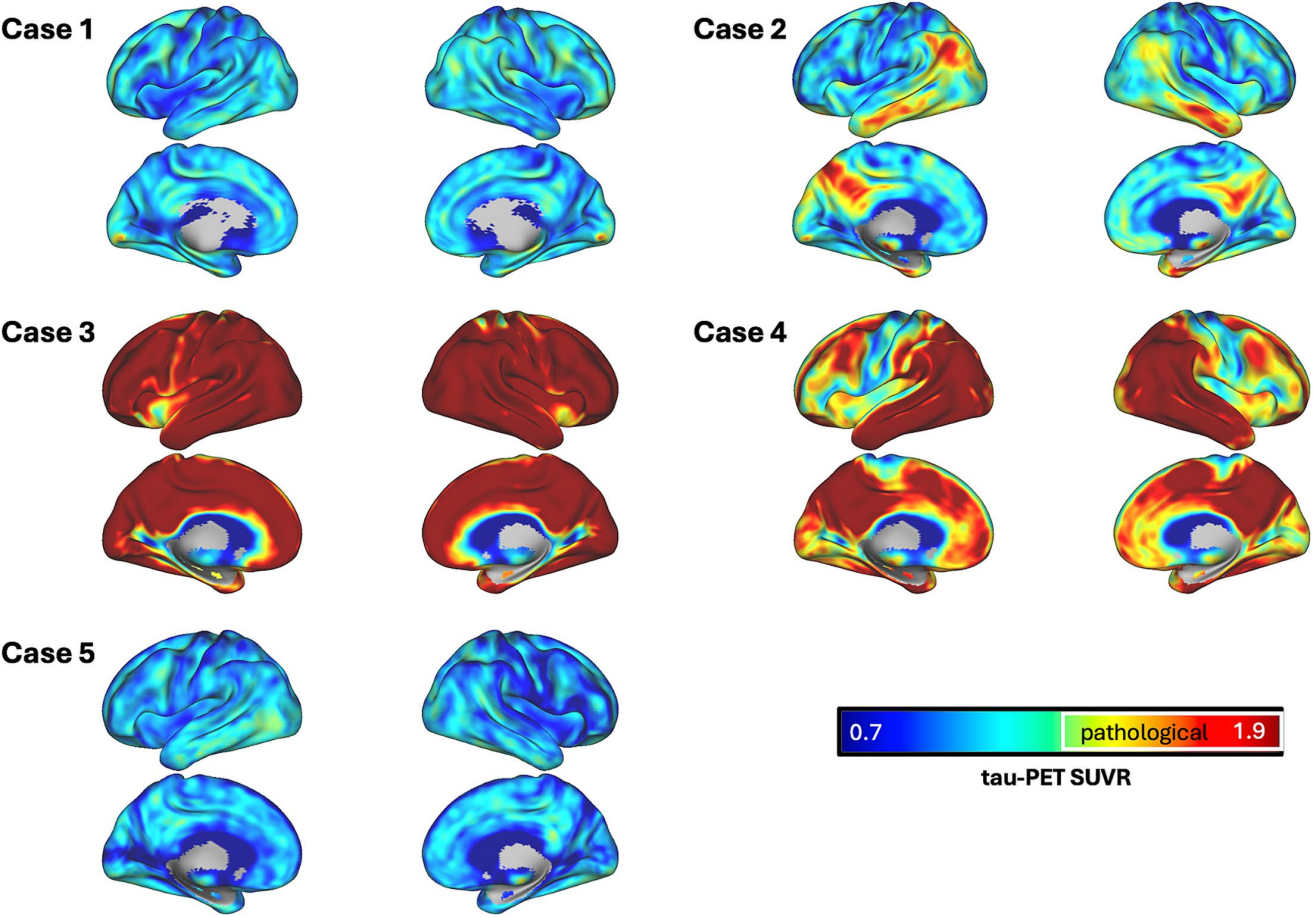
Surface renderings of SUVR images for each case. Tau PET SUVRs are shown as continuous values, white outlines define areas which surpass a pre-established pathological tau SUVR threshold of 1.3.

In addition to the diagnostic reads, visual interpretation of all generated [^18^F]PI-2620 tau PETs (Figure 1) from our patients with DS, as well as five HC for reference, were performed by three physicians blinded to the patients’ symptoms and clinical diagnosis. The results showed good inter-rater reliability between the three for all Braak Stages leveraging Fleiss’s kappa (I-III: kappa = 0.6, *p* < 0.001; Braak IV: kappa = 1, *p* < 0.001; Braak V: kappa = 0.8, *p* < 0.001; Braak VI: kappa = 1, *p* < 0.001). Specifically, 3 out of 5 patients showed increased uptake of [^18^F]PI-2620 in the predefined Braak regions, thereby receiving the overall rating of being tau-positive, with case 3 and 4 exhibiting marked uptake in Braak I-VI while case 2 showed marked uptake in Braak I-III and moderate tracer binding in remaining Braak regions. Cases 1 and 5 on the other hand were found to show no qualitative tracer uptake in any Braak stage but showed mild tracer binding in the basal ganglia.

Assessing sensitivity and specificity for DS vs. HC depending on the visual read of basal ganglia, raters evaluated all but one of the HC as showing no tracer binding in this area and found in their majority read all adults with DS to show binding in the basal ganglia. This resulted in a specificity of 0.8 and a sensitivity of 1 for the differentiation of DS and HC via basal ganglia visual read using the [^18^F]PI-2620 tracer.

## Discussion

In this case series, we demonstrate five cases of clinical and/or cognitive decline in adults with DS undergoing tau PET imaging for further differential diagnosis.

All five assessments were carried out after obtaining written consent and without any clinical complications, adverse events or compliance issues raised by patients or their care givers during or after the procedure. Visual reading of these PETs showed good inter-rater reliability and revealed two patients without any increased tracer binding in the respective areas, one individual with moderate and the two remaining participants with pronounced tracer binding up to Braak VI.

Tau PET imaging has great potential to improve differential diagnosis in adults with DS at risk of developing AD and exhibiting cognitive decline in a clinical setting. Established diagnostic processes applied in sporadic AD such as lumbar puncture, for one considered rather invasive and therefore an unfavorable procedure as seen for case 5, further provide no detailed, region-specific information about tau accumulation in the brain, rendering tau PET imaging, specifically when using second-generation tau tracers with reduced off-target binding, a powerful tool for correlating pathology with clinical symptoms and tracking disease progression. Its implementation is further a necessary step for a better inclusion of adults with DS in AD research and getting them trial-ready for future interventional studies with anti-amyloid or anti-tau agents.

Adults with DS have been reported to exhibit tau tracer binding according to Braak stages (Fleming et al., 2023; Hartley et al., 2022; Rafii et al., 2017), with a similar pattern as in euploid individuals with sporadic rather than autosomal-dominant AD (Mann et al., 1986; Rafii et al., 2017; Mueller et al., 2020; Wisch et al., 2024). While information on the association of tau PET burden with CSF tau markers in DS is scarce, and more research is needed to understand the temporal relationship between these biomarker modalities in that population, studies in euploid adults with and without cognitive impairment have found positive correlations between CSF tau measures and PET tau burden especially in the symptomatic and amyloid-positive individuals (Janelidze et al., 2020; Gordon et al., 2016). At the same time, investigators have found a certain disconcordance between CSF phosphorylated tau and PET tau burden for the prediction of cognitive decline, suggesting caution with the interchangeability of both modalities (Bucci et al., 2021).

In DS, a study in plasma has found pTau-217 and GFAP consistently positively correlated with increased tau tracer binding in the temporal region of amyloid-positive DS individuals (Janelidze et al., 2022), suggesting the potential for a multimodal approach for increased diagnostic accuracy and ability to monitor disease progression as well as contextualizing tau PET results within the current framework of AD criteria.

Further, correlation of tau PET burden and cognitive performance has been established consistently in sporadic AD (Bucci et al., 2021; Mueller et al., 2020) as well as DS-AD (Rafii et al., 2017; Grigorova et al., 2022), showing a worse performance on neuropsychological assessments with marked tracer binding. While isolated baseline assessments are subject to great inter- and intrasubject variability (Nübling et al., 2020), we found that individuals with a longitudinal decline on the CAMCOG-DS did in fact exhibit increased tracer binding up to Braak stage VI at the point of PET imaging. Accordingly, for case 1, exhibiting no objectifiable longitudinal decline in cognitive performance, our raters did not see any marked tracer binding according to Braak staging, suggesting a stable disease status. Similarly, case 5, being well beyond the expected age of symptom onset exhibited an performance on the CAMCOG-DS set in the medium range and upon follow-up, showed no significant cognitive decline in the assessment, with raters finding no qualitative tracer uptake. This suggested impairment in functional activities due to another reason than AD further to be investigated, however there was no amyloid status available.

We found amyloid PET burden associated with tau PET burden, whereas the CSF Aβ42/40 ratio did not consistently align with tau PET. For example, in case 1, neither tau nor amyloid PET showed significant tracer binding, yet the CSF Aβ42/40 ratio was notably decreased, suggesting a disconnect between CSF measures and PET findings. In contrast, case 2 demonstrated amyloid PET positivity, which was followed by a marked increase in tau PET tracer uptake 1 year later, highlighting the alignment between both measures. Further illustrating this pattern, case 3 showed extensive tau PET tracer binding despite normal Aβ42/40 levels (with only slightly reduced Aβ42). Case 4 did show a decreased Aβ42/40 ratio with positive tau PET findings. With prior studies reporting a strong correlation between amyloid and tau PET imaging (Zammit et al., 2021; Tudorascu et al., 2020; Zammit et al., 2024; Pegueroles et al., 2023), the discrepancy between CSF amyloid measures and tau PET could be due to the fact that the former change as early as about 25 years prior to symptom onset, while changes in tau metabolism are further down the temporal sequence of AD pathology as a direct result of manifest amyloid plaques appearing roughly 10 years after CSF amyloid changes (Fortea et al., 2020).

Considerable off-target binding to monoaminooxidases A and B has been widely reported for first-generation tracers, resulting in elevated signal in the basal ganglia and other structures (Ossenkoppele et al., 2018; Leuzy et al., 2019). With second-generation tracers such as [^18^F]PI-2620 this has been greatly reduced (Leuzy et al., 2019), allowing for a more reliable read-out of binding patterns, which becomes even more relevant considering that most but not all (Pegueroles et al., 2023; Nuebling et al., 2021) studies in DS so far have leveraged first-generation tau tracers, resulting in the need of the exclusion of the striatum due to apparent off-target binding (Hartley et al., 2022; Rafii et al., 2017; Grigorova et al., 2022; Zammit et al., 2021; Tudorascu et al., 2020; Handen et al., 2021). In our case series however, visual reads of the basal ganglia using [^18^F]PI-2620 demonstrated promising sensitivity and specificity for differentiating DS from healthy controls without AD and no clinical signs of cognitive impairment.

For the visual interpretation of tracer binding in the striatum, there remained a certain ambiguity which is represented in the lack of a majority read for two of the five individuals. The challenge of interpreting tau PET in AD in DS individuals as well as in other populations remains in the effort of distinguishing actual tau pathology from off-target binding, which underscores the importance of leveraging advanced tracers like [^18^F]PI-2620 with reduced off-target effects and high affinity to 3/4-repeat tau (Mueller et al., 2020). Therefore, we believe that this case series highlights the potential of [^18^F]PI-2620 to more easily overcome these limitations than earlier tracers, offering a foundation for future research to explore tau deposition in DS-AD with greater accuracy and reliability.

Another limitation lies in the small sample size which in turn limits the generalizability of our findings. Yet, this case study provides first valuable insights into the value of tau tracer [^18^F]PI-2620 in DS, which can serve as a foundation for larger, more comprehensive studies in the future.

In summary, we present the first qualitative analysis of the [^18^F]PI-2620 tau tracer in a case series of adults with DS and suspected cognitive decline, demonstrating its feasibility and practicability for clinical application. Our findings highlight the concordance between cognitive decline and increased cerebral tau tracer binding, underscoring its potential role in differential diagnosis within the context of DS-AD. Future research should focus on longitudinal imaging studies to further characterize disease progression, ideally leveraging the integration of tau and amyloid PET for a more comprehensive diagnostic framework, and aim to conduct comparative analyses across broader population groups.

## Data Availability

The datasets presented in this article are not readily available because before sharing, additional steps for de-identification and anonymization of patient information must be completed, and further approvals may be required. We are committed to making the data available after these necessary processes have been properly addressed. Requests to access the datasets should be directed to johannes.levin@med.uni-muenchen.de.
